# Klassevirus 1, a previously undescribed member of the family *Picornaviridae*, is globally widespread

**DOI:** 10.1186/1743-422X-6-86

**Published:** 2009-06-24

**Authors:** Lori R Holtz, Stacy R Finkbeiner, Guoyan Zhao, Carl D Kirkwood, Rosina Girones, James M Pipas, David Wang

**Affiliations:** 1 Department of Pediatrics, Washington University School of Medicine, One Children's Place, Campus Box 8116, St. Louis, Missouri 63110, USA; 2 Departments of Molecular Microbiology and Pathology and Immunology, Washington University School of Medicine, 660 S. Euclid Ave. Campus Box 8230, St. Louis, Missouri 63110, USA; 3 Enteric Virus Research Group, Murdoch Childrens Research Institute, Royal Children's Hospital, Victoria, Australia; 4 Department of Microbiology, Faculty Biology, University of Barcelona, Diagonal 645, 08028 Barcelona, Spain; 5 Department of Biological Sciences, University of Pittsburgh, 559B Crawford Hall, 4249 Fifth Avenue, Pittsburgh, Pennsylvania 15260, USA

## Abstract

**Background:**

Diarrhea is the third leading infectious cause of death worldwide and is estimated to be responsible for approximately 2 million deaths a year. While many infectious causes of diarrhea have been established, approximately 40% of all diarrhea cases are of unknown etiology. In an effort to identify novel viruses that may be causal agents of diarrhea, we used high throughput mass sequencing to analyze stool samples collected from patients with acute diarrhea.

**Results:**

Sequences with limited similarity to known picornaviruses were detected in a stool sample collected in Australia from a child with acute diarrhea. Using a combination of mass sequencing, RT-PCR, 5' RACE and 3' RACE, a 6383 bp fragment of the viral genome was sequenced. Phylogenetic analysis demonstrated that this virus was highly divergent from, but most closely related to, members of the genus *Kobuvirus*. We have tentatively named this novel virus klassevirus 1. We also detected klassevirus 1 by RT-PCR in a diarrhea specimen collected from a patient in St. Louis, United States as well as in untreated sewage collected in Barcelona, Spain.

**Conclusion:**

Klassevirus 1 is a previously undescribed picornavirus that is globally widespread and present on at least three continents. Further investigations to determine whether klassevirus 1 is a human pathogen are needed.

## Background

The impact of diarrhea is primarily felt in the developing world, where approximately 2 million deaths result from diarrhea annually [1-3]. In developed countries, where diarrhea related mortality is relatively rare, there is still nonetheless a tremendous disease burden. For example, in the United States, approximately 9% of all hospitalizations for children under age 5 years are due to diarrhea episodes [4]. While rotaviruses, caliciviruses, adenoviruses, and astroviruses are responsible for the greatest proportion of cases [5-8], approximately 40% of diarrhea cases are of unknown etiology [9-11].

Many picornaviruses can be detected in human stool such as enteroviruses, polio, Aichi virus, and cardioviruses [12-15]. Some of these viruses, such as Aichi virus, are associated with diarrheal disease [13] while others such as polio are shed fecally, but manifest pathogenicity in other organ systems. Picornaviruses are non-enveloped viruses with a single stranded positive-sense RNA genome that encodes a single polyprotein [12]. The picornavirus family currently consists of 14 proposed genera  associated with a diverse range of diseases. Viruses in six of these genera potentially infect humans (*Enterovirus*, *Hepatovirus*, *Parechovirus*, *Kobuvirus, Cosavirus*, and *Cardiovirus*). With the advent of culture independent molecular methods, many diverse new members of the picornavirus family have been identified in recent years. These include novel cardioviruses [14-17], rhinoviruses [18-20], parechoviruses [21,22] and the novel genus of cosaviruses [23,24]. These studies have demonstrated that significant viral diversity exists in the human gut that remains unexplored.

We have previously described a mass sequencing strategy based on high throughput Sanger sequencing to analyze human stool for previously undescribed viruses [25]. In this study, we used a similar strategy but incorporated a next-generation pyrosequencing platform (Roche Genome Sequencer) in place of traditional Sanger sequencing. This resulted in the identification of a highly divergent picornavirus in a stool sample collected in 1984 from a child in Australia with acute diarrhea. Sequencing and phylogenetic analysis demonstrated that this virus is a novel member of the family *Picornaviridae*. We propose that this virus be named klassevirus 1 (**k**obu-**l**ike virus **a**ssociated with **s**tool and **se**wage).

## Results

### Identification and sequencing of klassevirus 1

Extracted nucleic acid from a stool specimen collected in 1984 from a child with acute diarrhea was subjected to high throughput mass sequencing using 454 pyroseqencing technology. From the resulting reads, two sequences were identified by BLAST that had only limited sequence identity to known viruses. One was a 217 bp fragment that upon translation to amino acid sequence had 42% identity to its closest relative, Aichi virus (2B/2C region). The second sequence read of 443 bp had 40% amino acid identity to the VP0 region of Aichi virus. From these two initial sequences, a 6383 bp contig (klasse-mel1) was generated by RT-PCR and multiple 3' and 5' random amplification of cDNA ends (RACE) reactions. The 5' end of this contig aligned to the predicted VP0 protein at the N-terminus of the polyprotein and extended past the predicted 3D protein at the C-terminus of the polyprotein to the poly-A tail. The initial assembly was confirmed by sequencing multiple overlapping RT-products spanning the length of the contig to give 3.3X coverage. We were not able to extend the contig further in the 5' direction despite performing multiple 5' RACE reactions using different primers with multiple high temperature (70°C) reverse transcriptases (rTth [Applied Biosystems] and Thermoscript [Invitrogen]).

**Figure 1:**
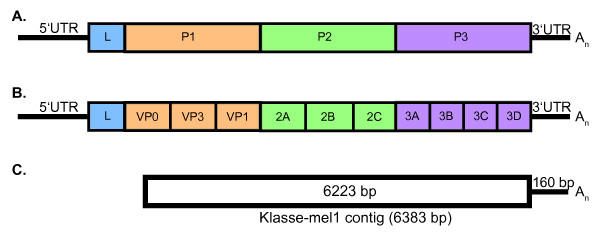
**Genomic organization of kobuviruses**. (A) Schematic of initial protein products P1, P2, and P3. (B) Schematic of processed polyprotein. (C) Representation of sequence obtained from klasse-mel1 virus.

The klassevirus 1 contig had a genomic organization similar to other picornaviruses (Figure 1A–C). Conserved Pfam [26] motifs characteristic of picornaviruses were present including two picornavirus capsid proteins, RNA helicase, 3C cysteine protease, and RNA dependent RNA polymerase.

### Phylogenetic analysis of klassevirus 1

**Figure 2:**
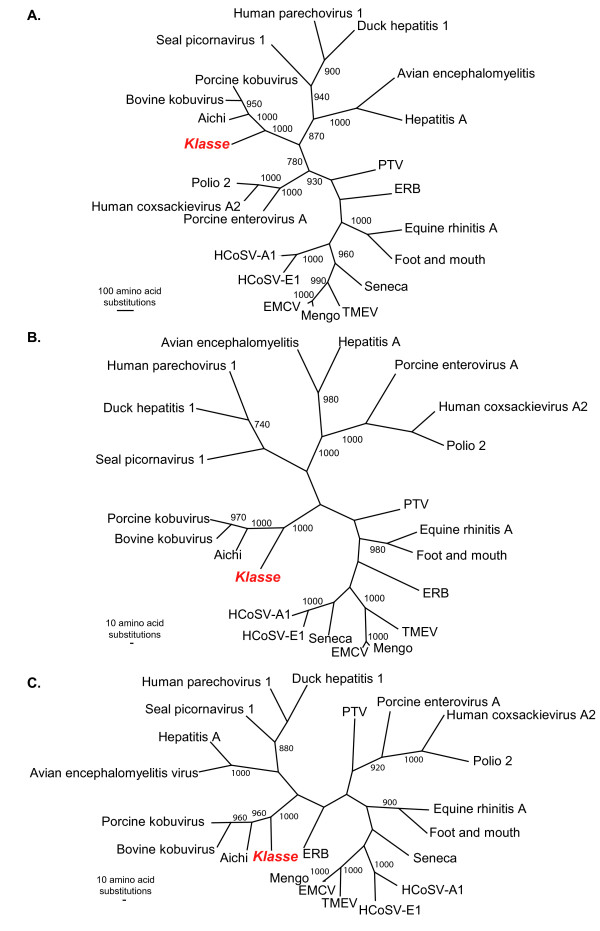
**Phylogenetic anaylsis of klassevirus 1 sequence**. Multiple sequence alignments were generated with klassevirus 1 and the corresponding regions of known picornaviruses using ClustalX. (A) P3 region, (B) P2, and (C) VP3/VP1. Paup was used to generate maximum parsimony phylogenetic trees. Bootstrap values > 700 from 1,000 replicates are shown. PTV: Porcine teschovirus; ERB: Equine rhinitis B virus.

Phylogenetic analysis of the VP3/VP1, P2, and P3 regions of the genome demonstrated that this virus sequence is highly divergent from all previously described picornaviruses (Figure 2A–C). The closest relatives of klassevirus 1 appeared to be members of the genus *Kobuvirus*, which includes Aichi virus, bovine kobuvirus and porcine kobuvirus.

### Epidemiological survey for klassevirus 1

In order to determine the prevalence of klassevirus 1, two patient cohorts were examined by RT-PCR. In the first cohort, 340 pediatric stool specimens sent to the clinical microbiology lab for bacterial culture at the St. Louis Children's Hospital in St. Louis MO, USA were tested. One sample from this cohort was positive by RT-PCR. The amplicon from this patient's virus (designated klasse-stl1) was found to have 92% identity at the amino acid level and 88% nucleotide identity to the original klasse-mel1 contig. Screening of 143 stool samples from children with acute diarrhea collected from the Royal Children's Hospital (Melbourne, Australia) did not yield any additional positive samples. In addition, RT-PCR was performed on concentrated raw sewage collected in Barcelona, Spain. A PCR product of the expected size was obtained and directly sequenced. This fragment (klasse-bar1) shared 85% amino acid identity and 84% nucleotide identity to the original klasse-mel1 sequence (Table 1).

**Table 1:** Pair-wise amino acid identities between three klassevirus 1 strains using partial sequence fragments.

Sequence Fragment	Klasse-stl1	Klasse-bar1	Klasse-mel1
Klasse-stl1 3D	100%	97%	96%

Klasse-slt1 2C	100%	87%	92%

Klasse-stl1 VP0/VP3	100%	91%	96%

Klasse-bar1 3D		100%	97%

Klasse-bar1 2C		100%	85%

Klasse-bar1 VP0/VP3		100%	91%

Klasse-mel1 3D			100%

Klasse-mel1 2C			100%

Klasse-mel1 VP0/VP3			100%

To further compare the divergence between the three positive samples of klassevirus 1, RT-PCR was performed using primers that target the polymerase region (3D) of the genome and the VP0/VP3 region. Approximately one kb of additional sequence was generated from klasse-bar1 and klasse-stl1 in both of these regions (see methods). Pair-wise amino acid identities ranged from 85%–97%, with the greatest degree of sequence conservation in the 3D region (Table 1).

## Discussion

In this study, we identified a previously undescribed picornavirus present in stool and sewage. Phylogenetic analysis demonstrated that this virus is most closely related to other picornaviruses in the genus *Kobuvirus*. Based on the criteria established by the picornavirus study group, members of a genus should share > 40%, > 40% and > 50% amino acid identity in P1, P2 and P3 genome regions respectively [27]. Klassevirus 1 shared only 43% amino acid identity in the P3 region and 33% amino acid identity in the P2 region to its closest relative, Aichi virus. Given these observations, and using strictly the percent identity definitions, klassevirus 1 may represent the first member of new picornavirus genus. However, we note that at all loci, bootstrap analysis suggests that klassevirus 1 diverged from an ancestor common to all of the known kobuviruses. Thus the formal classification of klassevirus 1 at the genus level is currently uncertain and subject to further discussion per the ICTV.

Subsequent screening by RT-PCR using primers targeting the 2C region of the genome established that klassevirus 1-like sequences were present not only in Australia, but also in North America and Europe. The presence of klassevirus 1 in the United States was determined by the traditional strategy of screening of individual stool samples. In addition, we also examined raw sewage collected in Barcelona to see if we could detect klassevirus 1. Sewage represents a pooled meta-sample of literally thousands of individual specimens. Known enteric viruses such as adenoviruses [28,29], noroviruses [30] astroviruses [31], and hepatitis A [32] have frequently been tested for and detected in sewage by PCR and RT-PCR. We reasoned that detection of klassevirus 1 in raw sewage would serve as a proxy for its presence in human stool in the population that generated the sewage. Since the exact history of the sewage is poorly defined, it is possible that other waste products, such as animal feces could contribute to the raw sewage meta-genome. Nonetheless, we propose that raw sewage screening from a diversity of sites can serve to rapidly define the geographic distribution of a given virus. The detection of klassevirus 1 in stool and sewage from Melbourne, Barcelona and St. Louis, demonstrates that klassevirus 1 is globally distributed. Moreover, since both the Barcelona sewage and St. Louis stool specimens were collected in 2008, we conclude that klassevirus 1 is currently circulating in the human population.

Whether klassevirus 1 represents a true human pathogen remains to be determined. It is possible that klassevirus 1 is a human pathogen that causes gastroenteritis. It is also possible that klassevirus 1 injures other organs but is excreted through the intestinal tract like poliovirus. Another possibility is that klassevirus 1 is a human commensal virus. Alternatively, klassevirus 1 could represent a non-human virus acquired from dietary exposure. Further investigations are needed to determine if klassevirus 1 is a causal agent of human disease(s). To begin addressing this question, epidemiologic studies including case-control and seroprevalence analyses are needed.

## Materials and methods

### Primary Stool Specimen

This stool was collected in 1984 from a 38 month old child presenting to the emergency department of the Royal Children's Hospital, Melbourne, Australia with acute diarrhea and stored at -80°C. Previous testing of this diarrhea specimen for known enteric pathogens using routine enzyme immunoassays (EIA) and culture assays for rotaviruses, adenoviruses, and common bacterial and parasitic pathogens was negative [6]. Additionally, RT-PCR assays for caliciviruses and astroviruses were also negative [6,33].

### Sample preparation for 454 sequencing

120 mg of frozen stool was chipped and then resuspended in 6 volumes of PBS [25]. The sample was centrifuged to pellet particulate matter and the supernatant was then passed through a 0.45 μm filter. Total nucleic acid was isolated from 100 μL primary stool filtrate using QiAmp DNA extraction kit (Qiagen) according to manufacturer's instructions. Total nucleic acid was randomly amplified using the Round AB protocol as previously described [34]. This was then pyrosequenced on a Roche FLX Genome sequencer (Roche) according to manufacturer's protocol. To eliminate sequence redundancy in each library sequences were clustered using BLASTCLUST from the 2.2.17 version of NCBI BLAST. Sequences were clustered based on 98% identity over 98% sequence length and the longest sequence from each cluster was chosen as the representative sequence of the cluster. Unique sequences were filtered for repetitive sequences and then compared with the GenBank nr database by BLASTN and TBLASTX.

### Sequencing of klassevirus 1

For sequencing experiments, the stool filtrate was proteinase K treated prior to RNA extraction. RNA was isolated from primary stool filtrate using RNA-Bee (Tel-Test, Inc.) according to manufacturer's instructions. RT-PCR and 3'RACE reactions were performed using SuperScript III and Platinum Taq (Invitrogen One-Step RT-PCR). For 5'RACE reactions cDNA was generated with Themoscript (Invitrogen) and amplified with Accuprime Taq (Invitrogen). Amplicons were either cloned into pCR4 (Invitrogen) or sequenced directly.

### Phylogenetic Analysis

Protein sequences associated with the following reference virus genomes were obtained from GenBank: Equine Rhinitis A virus (NP_653075.1), Foot-and-mouth-type-O (NP_658990.1), Equine Rhinitis B virus (NP_653077.1), Theiler murine encephalomyelitis (AAA47929.1), Mengo virus (AAA46547.1), Encephalomyocarditis virus (CAA60776.1), Seneca valley virus (DQ641257), Aichi virus (NP_047200.1), Porcine teschovirus (NP_653143.1), Human Cosavirus E-1 (FJ555055.1), Hepatitis A Virus (M14707), Bovine kobuvirus (NP_740257.1), Porcine kobuvirus (YP_002456506.1), Human coxsackievirus A2 (AAR38840.1), Porcine enterovirus A (NC_003987), Human poliovirus 2 (M12197), Avian encephalomyelitis virus (NC_003990), Duck hepatitis virus 1 (ABI23434), Seal picornavirus type 1 (NC_009891), Human Cosavirus A1 (FJ438902), and Human parechovirus 1 (AAA72291.1). Multiple sequence alignments were performed using ClustalX (1.83). The amino acid alignments generated by ClustalX were input into PAUP [35], and maximum parsimony analysis was performed using the default settings with 1,000 replicates.

### Epidemiological survey for klassevirus 1

#### Melbourne Cohort

Stool samples were collected from children under the age of 5 who were admitted to the Royal Children's Hospital, Melbourne, Victoria, Australia with acute diarrhea between 1978 and 1999. For a portion of these samples (70), RNA was extracted in the same manner as the primary sample. For the remaining specimens (73), chips of frozen fecal specimens (~30–150 mg) were resuspended in 6 volumes of PBS. Total nucleic acid was extracted from 200 μL of each stool suspension using a MagnaPure LC instrument (Roche). 200 μL of water was used to elute the total nucleic acid from each sample.

#### St. Louis Cohort

Leftover material from 340 stool specimens that were routinely submitted to the St. Louis Children's Hospital Lab for bacterial culture were collected from January 2008–July 2008. For these specimens total nucleic acid was extracted as described above. This study was approved by the Human Research Protection Office of Washington University.

#### Raw sewage

One 10 L-sample of raw sewage was collected in an urban wastewater treatment plant in the area of Barcelona, Spain. The sample was collected in a sterile container and stored for up to 2 hours at 4°C before being processed. The viruses present in the sample were concentrated in 30 mL of phosphate buffer by organic flocculation based on the procedure previously described by Calgua et al., 2008 [36]. A second concentration step with elution of the viral particles was performed. Briefly, 10 mL of the viral concentrate were eluted with 40 mL of 0.25 M glycine buffer (pH 9,5) at 4°C, suspended solids were separated by low speed centrifugation at 7500 × g for 30 min at 4°C and the viruses present in the supernatant were finally concentrated in 1 mL of PBS by ultracentrifugation at 87500 × g for 1 h at 4°C. This was then DNase treated, and then total nucleic acid was extracted.

#### RT-PCR for detection of klassevirus 1

Primers expected to generate a 345 bp product were designed to the 2C region of klassevirus 1 (LG0098: 5'-CGTCAGGGTGTTCGTGATTA-3' and LG0093: 5'-AGAGAGAGCTGTGGAGTAATTAGTA-3'). RT-PCR reactions were performed using Qiagen one-step kit under the following conditions: 30 min RT step, 94°C hold for 10 min, followed by 40 cycles of 94°C for 30 s, 56°C for 30 s, and 72°C for 60 s. In order to further compare strain divergence, primers expected to produce amplicons of 1001 bp and 1025 bp based on the klasse-mel1 sequence were designed targeting the 3D and VP0/VP3 regions, respectively: (LG0118: 5'-ATGGCAACCCTGTCCCTGAG-3' and LG0117 5'-GGAAACCCAACCACGCTGTA-3') and (LG0119: 5'-GCTAACTCTAATGCTGCCACC-3' and LG0136: 5'-GCTAGGTCAGTGGAAGGATCA-3'). These RT-PCR reactions were performed using the Invitrogen One-Step RT-PCR kit with the following conditions: 30 min RT step at 60°C, 94°C hold for 2 min, followed by 40 cycles of 94°C for 15 s, 56°C for 30 s, 68°C for 90 s. Whenever possible, amplicons were cloned into pCR4 (Invitrogen) and sequenced using standard Sanger sequencing technology. In some instances, PCR products were directly sequenced and only high quality sequence from those samples were included in analysis. All klassevirus 1 sequences have been deposited in Genbank (GQ253930-GQ253936).

## Competing interests

The authors declare that they have no competing interests.

## Authors' contributions

LH performed the experiments, sequence analysis and wrote the paper. SF and GZ performed sequence analysis; CK, RG and JP provided stool and sewage for analysis, DW conceived the project and helped write the paper. All authors have read and approved the final manuscript.
